# The efficacy of iatrosedation and music listening techniques in attenuating dental anxiety in patients undergoing dental crown preparation: A randomized clinical trial

**DOI:** 10.1002/pchj.731

**Published:** 2024-02-16

**Authors:** Abhishek Lal, Afsheen Maqsood, Naseer Ahmed, Sara Altamash, Mohammed Q. Al Rifaiy, Rawan Alsaif, Fahim Vohra, Seyed Ali Mosaddad, Artak Heboyan

**Affiliations:** ^1^ Department of Prosthodontics Altamash Institute of Dental Medicine Karachi Pakistan; ^2^ Department of Oral Pathology Bahria University Dental College Karachi Pakistan; ^3^ Department of Orthodontics Altamash Institute of Dental Medicine Karachi Pakistan; ^4^ Department of Prosthetic Dental Science, College of Dentistry King Saud University Riyadh Saudi Arabia; ^5^ Student Research Committee, School of Dentistry Shiraz University of Medical Sciences Shiraz Iran; ^6^ Department of Research Analytics, Saveetha Dental College and Hospitals Saveetha Institute of Medical and Technical Sciences, Saveetha University Chennai India; ^7^ Department of Prosthodontics, Faculty of Stomatology Yerevan State Medical University after Mkhitar Heratsi Yerevan Armenia

**Keywords:** anxiety, dental treatment, iatrosedation, music listening, patient perception

## Abstract

Dental anxiety is a common problem encountered in dental clinics that affects both patients and dentists. Adequate management of dental anxiety is critical for optimal treatment outcomes for the patient. This study aims to assess the efficacy of two anxiety‐reduction techniques (iatrosedation and music listening) for dental crown preparation in adult patients. In this clinical trial, 60 patients were randomly assigned to three groups: Group 1, iatrosedation; Group 2, music listening; and Group 3, control. Patients in all three groups underwent dental crown preparation. To measure the anxiety levels of the patients, heart rate was calculated using a pulse oximeter, and verbal rating scale scores were assessed. One‐way analysis of variance, post hoc analysis, and Spearman's correlation were used to compare the mean values of the three groups. Significant differences were observed in the heart rate and verbal rating scale scores among individuals in the study groups. A more substantial reduction in anxiety levels was found in patients exposed to iatrosedation (Group 1), which was followed by music listening (Group 2). Recorded heart rate and verbal rating scores were the highest in the control group patients. The iatrosedation technique significantly reduced dental anxiety for patients undergoing dental crown treatment; however, music listening was less effective than iatrosedation. Educating patients regarding the dental care they are about to receive is vital for reducing their anxiety.

## INTRODUCTION

A commonly encountered factor that affects both the patient and the dentist during dental procedures is dental anxiety. Anxiety can significantly compromise patients' access and utilization of oral health care, leading to oral disease, episodes of pain, swelling, and difficulty in masticatory function and, therefore, to poor quality of life (Yildirim, 2016). Patients who suffer from dental anxiety mainly experience systematic symptoms such as sweating, palpitation, increased heart rate and blood pressure, nausea, and hyperventilation (Saatchi et al., 2015). Both adult and pediatric patients suffer from mild to severe dental anxiety, with up to 16% prevalence among adults (Enkling et al., 2006; Pohjola et al., 2016).

A patient's first dental experience determines their cooperation and level of comfort, as painful and distressing experiences lead to avoidance behavior. Treatment avoidance by patients owing to dental anxiety leads to delayed and missed appointments, with a late visit to the dentist when an emergency arises. Furthermore, a high level of anxiety makes the treatment an unpleasant and stressful experience for both the patient and the dentist. Therefore, managing a patient's stress levels is paramount for any dental treatment to achieve optimal outcomes. Patients who avoid visiting the dentist suffer from their oral health deteriorating, with problems such as dental caries, gingivitis, periodontitis, and halitosis commonly found in such patients (Griffin et al., 2012).

For treatment success, patient cooperation during dental treatment is of paramount importance, and management of dental anxiety is critical. Various techniques are proposed and employed in clinical practice to alleviate dental anxiety, including distraction, hypnosis, breathing exercises, and analgesia (inability to feel pain) (De Jongh et al., 2005). One such method is iatrosedation, a psychosedative technique introduced by Friedman in 1998 (Friedman & Wood, 1998). Iatrosedation comprises the iatrosedative interview followed by the clinical encounter. In the iatrosedative interview, communication with the patient starts with verbal and non‐verbal methods to identify the patient's fears, followed by formulation of a diagnosis and treatment plan (Friedman & Wood, 1998). The second part of iatrosedation is a clinical encounter in which the entire treatment plan is thoroughly explained to the patient, questions are answered, and the treatment is performed (Friedman & Wood, 1998).

Another contemporary technique to manage patient stress during dental visits is music listening. Music interventions have been used as an alternative to pharmacological methods of anxiety management in patients (Bradt et al., 2013). Music listening, also called music medicine, involves an individual listening to their chosen pre‐recorded music (Graff, 2017). Music listening differs from music therapy, where credentialed music therapists use music that collaborates with patients regarding their individual needs (Bradt & Teague, 2018; Graff, 2017). Compared with music therapy, music listening is a more frequently preferred intervention for patients undergoing dental treatment (Bradt & Teague, 2018). Several studies in the literature have explored the effects of music listening on reducing the anxiety levels of patients (Ainscough et al., 2019; Chandure et al., 2017; Pande et al., 2017; Singh et al., 2014). When a possible threat is perceived, fear results in the activation of the sympathetic nervous system (fight or flight system), which releases hormones such as epinephrine and norepinephrine that generate anxiety symptoms (Yoshihara et al., 2016). Listening to music is known to alleviate various kinds of anxiety experienced by individuals in relation to examinations, medical treatment, and sports competitions (Yoshihara et al., 2016). In addition, auditory music listening decreases the patient's heart rate, blood pressure, and cortisol levels (Koelsch et al., 2016; Linnemann et al., 2015). Music listening is known to reduce anxiety levels by effectively decreasing the activation of the sympathetic nervous system and, at the same time, increasing the activity of the parasympathetic nervous system (Wu et al., 2020).

Besides music listening and iatrosedation, various techniques have been employed to reduce the level of anxiety of patients. Pharmacological therapy has been successfully used extensively, especially in children, and consists of various sedative drugs such as midazolam and ketamine (Gazal et al., 2015; Gazal, Fareed, et al., 2016). The most commonly utilized drugs to relieve anxiety are benzodiazepines, including clonazepam, lorazepam, and alprazolam. Selective serotonin reuptake inhibitors are also widely used to treat anxiety, including dental anxiety; however, benzodiazepines remain the preferred option.

In addition to pharmacological, non‐pharmacological modalities, including verbal and visual approaches, have also been utilized to reduce dental anxiety (Gazal et al., 2015). This study found that patients reported decreased anxiety levels using visual methods compared with verbal ones (Gazal, Tola, et al., 2016). Non‐pharmacological modalities should always be exhausted before opting for pharmacological therapies. Some non‐pharmacological modalities include behavior management techniques and cognitive behavior therapy (CBT). CBT has many uses for addressing the psychological challenges faced by different types of patient, with its use having been studied in dental anxiety. CBT, as a technique to reduce dental anxiety, has been successfully used to treat dental anxiety in children (Bux et al., 2019).

Iatrosedation is commonly practiced for anxiolysis; however, the evidence to support its clinical application is minimal. In one study (Friedman & Wood, 1998), the efficacy of iatrosedative interviews compared with standard interviews among dental‐anxiety patients using the Corah dental anxiety scale was assessed. They concluded that the iatrosedative interview was more effective in reducing dental anxiety than standard patient interviews (Friedman & Wood, 1998). Previously used anxiety‐reduction techniques focus primarily on successfully performing the treatment on the patient, not in helping patients overcome their dental anxiety (Taneja, 2015). However, recent evidence on the comparative efficacy of iatrosedation and music listening on anxiety reduction in dental patients is lacking. It is hypothesized that both iatrosedation and music listening will lead to significant anxiety reduction in patients treated for dental crowns as compared to controls. Therefore, the present study aimed to assess the comparative efficacy of iatrosedation and auditory mood induction (music listening) in reducing dental anxiety levels in adult patients undergoing dental crown preparations.

## METHODS

### Study design and sample size

This study was designed as a single‐blinded, randomized, controlled trial comprising three parallel groups. The study was executed as per the Declaration of Helsinki with the approval of the Ethics Review Committee of Altamash Institute of Dental Medicine, Pakistan (AIDM/ERC/08/2021/04). This study was carried out between August 2021 and January 2022 in the Department of Prosthodontics, Altamash Institute of Dental Medicine, Pakistan. OpenEpi software, AG Dean, KM Sullivan, MM Soe, USA (version 3.01) calculated the sample size, keeping the confidence interval at 95% and the desired percentile at 50. The sample size was calculated to be 20 patients per group (a total of 60 patients). The trial is registered on clinicaltrial.gov (trial number NCT: 05755126).

### Inclusion and exclusion criteria

**FIGURE 1:**
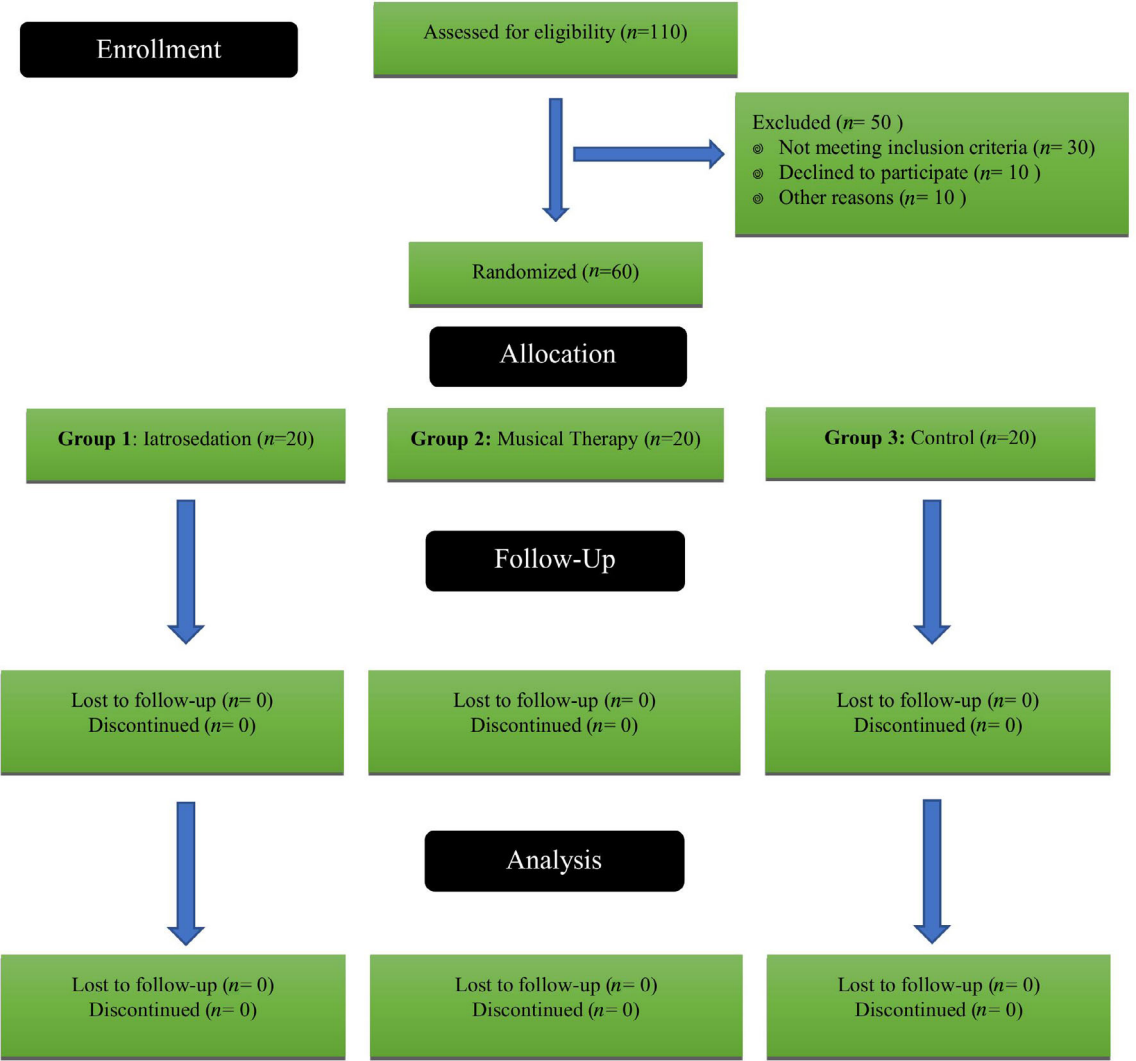
CONSORT flow diagram of the study.

Regarding the inclusion criteria, patients above 18 years of age, who had no systemic diseases, had no previous dental treatment experience, and showed a willingness to participate in the study were included. Patients under 18, medically compromised, and with a prior history of dental treatment were excluded from this study. A total of 110 patients visited the outpatient department, out of which 50 were excluded based on the study's exclusion criteria. The patient inclusion and follow‐up process is presented in Figure 1, following Consolidated Standards of Reporting Trials (CONSORT) guidelines.

### Study participants

After the demographic data were collected from the patients, 60 patients were divided into three groups with a ratio of 1:1:1, by randomization (lottery method), with each group having 20 patients as follows: Group 1, iatrosedation; Group 2, music listening; and Group 3, control. Patients' pulse and heart rate were measured before their crown preparation appointment using a pulse oximeter (Finger Pulse Oximeter YP‐1, A&D Medical, San Jose, California, USA ©) to assess anxiety levels. In addition, a verbal rating scale (VRS) was used to measure anxiety levels, which consisted of a scale from 0 to 10, with a score of 0 representing “*no distress*” and a score of 10 representing “*severe distress*.” All the patients belonging to the three groups underwent crown preparation, and their before and after treatment anxiety levels were evaluated using a pulse oximeter and VRS score.

### Study interventions

For the iatrosedation group, the first part of iatrosedation involved an iatrosedative interview consisting of identifying the patient's specific fears and acknowledging such fears, exploring and recognizing their intensity, and explaining the dental problem and treatment to the patient. The second part consisted of clinical encounters, where patients' fears are addressed clinically by answering questions before the commencement of treatment.

For the music‐listening group, patients were asked for their favorite music. Upon the patient's request, their favorite music was played, after confirming they could hear it clearly with headphones. The sound level was adjusted according to the patient's requirements so that they could easily listen to it without any disturbance. Once it was time to begin the dental procedure, the headphones were taken off, and the patient was then instructed to be seated in the dental unit to commence their treatment. For the control group, no intervention was performed, and the patients received their dental treatment.

### Procedure

The entire procedure was performed by a single dentist (AL). The dentist (AL) started by measuring the patients' parameters, including heart rate and VRS scores, before performing the study intervention, according to the different groups. After recording these parameters, patients were administered local dental anesthesia using a short 27‐gauge needle (Septodont‐Septoject Needle, Universal Dental (PVT) LTD, Saint‐Maur‐des‐Fosses, Paris, France  ©) consisting of 1:80,000 lidocaine solution with epinephrine by the principal investigator (AL). Crown preparation was then commenced (by AL). At the end of the procedure, heart rate and VRS were again recorded.

### Statistical analysis

Statistical Program for the Social Sciences (SPSS version 25, IBM, NY, USA) was employed for the data analysis. The anxiety levels in each of the three groups, recorded using a pulse oximeter and VRS before and after treatment, were compared. Descriptive statistics and ANOVA tests were utilized to compare the mean values obtained from each group to compare their effectiveness. Post hoc (Tukey test) analysis was performed for the inter‐group comparison of anxiety levels. To assess the effect of age and sex on anxiety levels, Spearman's correlation was performed. A *p*‐value of ≤ .050 was considered statistically significant.

## RESULTS

The distribution of males and females in each group was as follows: Group 1, 11 and 9; Group 2, 10 and 10; and Group 3, 9 and 11, respectively. The mean age of patients in each group was as follows: Group 1, 38.85 ± 10.95 years; Group 2, 35.25 ± 12.32 years; and Group 3, 36.20 ± 11.08 years.

**FIGURE 2:**
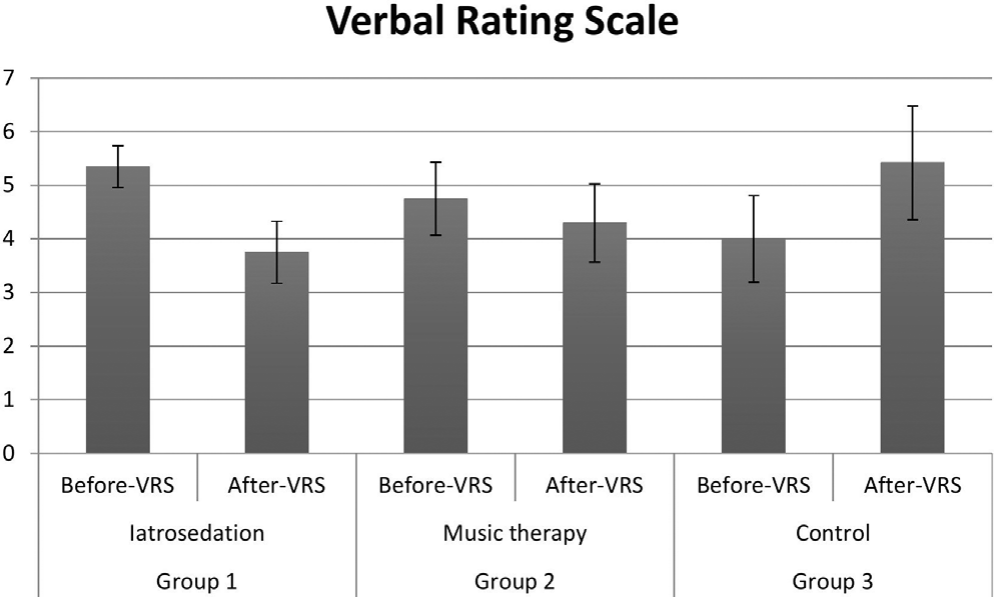
Comparison of verbal rating scale scores between study groups.

The mean heart rate before intervention among patients was 93.85 ± 4.84 BPM (Group 1), 89.95 ± 5.11 BPM (Group 2), and 82.10 ± 5.35 BPM (Group 3, control). Patients in Group 1 (subjected to iatrosedation) showed the maximum reduction in heart rate, which was statistically significant (*p* < .040) (Table 1). Subjects in Group 2 showed a decrease in heart rate before and after intervention (music listening); however, it was statistically insignificant (*p* > .050), and control subjects showed an increase in heart rate due to dental treatment. A statistically significant difference was noted in heart rates between patients belonging to the three groups (*p* = ≤ .001); furthermore, using post hoc analysis, the reduction of heart rate was reported to be higher in Group 1, followed by Group 2; however, Group 3 showed an increase in heart rate, as presented in Table 1 (Figure 2). Additionally, a significant difference was found in before and after heart rates using paired *t* tests in Group 1 (*p* = .001), Group 2 (*p* = .001), and Group 3 (*p* = .001), as presented in Table 2.

**TABLE 1:** Comparison of heart rate among the groups.

Group		*n*	Mean	Standard deviation	*p*‐value
Group 1 Iatrosedation	Before heart rate	20	93.85	4.85	.040
	After heart rate	20	85.30	5.41	
Group 2 Music listening	Before heart rate	20	89.95	5.11	.999
	After heart rate	20	88.75	6.15	
Group 3 Control	Before heart rate	20	82.10	5.35	.061
	After heart rate	20	89.50	6.79	

**TABLE 2:** Comparison of heart rates among the three study groups (*N* = 60).

Anxiety‐reduction technique		*n*	Mean	Standard deviation	*p*‐value
Group 1 Iatrosedation	Before heart rate	20	93.85	10.84	.001
	After heart rate	20	85.30	7.41	
Group 2 Music listening	Before heart rate	20	89.95	11.11	.001
	After heart rate	20	88.75	13.15	
Group 3 Control	Before heart rate	20	82.10	5.35	.001
	After heart rate	20	89.50	9.79	

The comparison of VRS scores among individuals in the study groups is presented in Table 3. Before implementing anxiety‐reduction techniques, the VRS scores of patients in groups 1, 2, and 3 were 5.35 ± 0.39, 4.75 ± 0.68, and 4.00 ± 0.81, respectively. Amongst the three groups, patients in Group 1 showed the maximum reduction in VRS score (1.6 ± 0.48), which was statistically significant (*p* < .050) (Figure 2). Subjects in Group 2 showed a low reduction in VRS score (0.45 ± 0.70) compared with Group 1, which was statistically insignificant (*p* > .050). Patients in Group 3 (control) showed an increase in VRS score (1.42 ± 0.93); this increase was not statistically significant (*p* > .050). A significant difference was observed in the VRS scores among the study groups (*p* = .034). Regarding the comparison of VRS scores before and after anxiety‐reduction techniques using the Wilcoxon sign‐rank test, a significant difference was found in Group 1 (*p* = .002) and Group 3 (*p* = .002), as presented in Table 4. Using the Kruskal–Wallis test, it was noted that a significant difference was found (*p* = .035) in the comparison of before and after VRS scores among the study groups, as presented in Table 5. Moreover, using the Mann–Whitney U test to compare the VRS scores between the groups, a significant difference was noted in Group 1 (*p* = .027) and Group 3 (*p* = .033), as presented in Table 6.

**TABLE 3:** Comparison of VRS scores among the groups (post hoc).

Anxiety reduction technique		Mean	Standard deviation	*p*‐value
Group 1 (Iatrosedation)	Before VRS	5.35	0.39	.011
	After VRS	3.75	0.58	
Group 2 (Music listening)	Before VRS	4.75	0.68	.810
	After VRS	4.30	0.73	
Group 3 (Control)	Before VRS	4.00	0.81	.700
	After VRS	5.42	1.06	

Abbreviation: VRS, verbal rating scale.

**TABLE 4:** Comparison of VRS scores among the three groups (*N* = 60).

		Mean rank	Sum of ranks	Z	*p*‐value
Iatrosedation after VRS – Iatrosedation before VRS	Negative ranks	10.43	156.50	−3.126	.002
	Positive ranks	4.83	14.50		
Music listening after VRS – Music listening before VRS	Negative ranks	7.11	64.00	0.211	.833
	Positive ranks	10.29	72.00		
Control after VRS – Control before VRS	Negative ranks	7.17	21.50	−3.167	.002
	Positive ranks	11.09	188.50		

Abbreviation: VRS, verbal rating scale.

**TABLE 5:** Comparison of before and after VRS scores among the three study groups using the Kruskal–Wallis test (*n* = 60).

Variables	Groups	*n*	Mean rank	Kruskal–Wallis	*p*‐value
Before VRS scores	Iatrosedation	20	35.53	4.316	.116
	Music	20	31.58		
	Control	20	24.40		
After VRS scores	Iatrosedation	20	23.35	6.701	.035
	Music	20	30.68		
	Control	20	37.48		

Abbreviation: VRS, verbal rating scale.

**TABLE 6:** Comparison of before and after VRS scores among the three study groups using the Mann–Whitney U test (*N* = 60).

Groups	*n*	Mean rank	Sum of ranks	Mann–Whitney U	*p*‐value
Iatrosedation before VRS	20	24.53	490.50	329.50	.027
Iatrosedation after VRS	20	16.48	329.50		
Music listening before VRS	20	20.88	417.50	192.50	.841
Music listening after VRS	20	20.13	402.50		
Control before VRS	20	16.58	331.50	121.50	.033
Control after VRS	20	24.43	488.50		

Abbreviation: VRS, verbal rating scale.

The comparison of the effect of age and sex on the anxiety levels of the individuals among the three groups is presented in Table 7. For subjects in Group 1, both age and sex were significantly related to VRS scores, with *p*‐values of .004 and .039, respectively. However, Group 2 subjects showed a significant age relation with heart rate (*p* = .033) and VRS (*p* = .019). Age and sex did not correlate with control subjects' heart rates and VRS scores.

**TABLE 7:** Comparison of age and sex with heart rates and VRS scores among the study groups.

Variable		Heart rate before intervention	Heart rate after intervention	VRS score before intervention	VRS score after intervention
Group 1 (Iatrosedation)					
Age	Correlation coefficient	−0.656	−0.422	−0.422	−0.617
	*p*‐value	.002	.64	.064	.004*
Sex	Correlation coefficient	0.428	0.420	0.467	0.465
	*p*‐value	.060	.066	.038	.039*
Group 2 (Music listening)					
Age	Correlation coefficient	−0.677	−0.477	−0.563	−0.519
	*p*‐value	.001	.033	.010	.019*
Sex	Correlation coefficient	0.121	0.017	−0.079	−0.175
	*p*‐value	.610	.942	.739	.459
Group 3 (Control)					
Age	Correlation coefficient	−0.423	−0.219	−0.281	−0.206
	*p*‐value	.063	.353	.231	.384
Sex	Correlation coefficient	0.096	0.149	0.082	0.239
	*p*‐value	.687	.532	.732	.309

Abbreviation: VRS, verbal rating scale.

* Showing significant correlation (*p* < .050).

## DISCUSSION

The present study aimed to evaluate the efficacy of iatrosedation and music‐listening techniques in dental anxiety reduction among patients treated for dental crowns. It was observed that only iatrosedation showed a significant decrease in anxiety compared with control subjects. Therefore, the study hypothesis was rejected. Dental anxiety is one of the most critical factors for both patients and dentists in achieving optimum treatment goals. Failure to address the anxious patient can result in a distressing treatment experience for the patient and a lack of treatment utilization, compromising oral health and quality of life.

In the present study, two parameters were assessed (heart rate and VRS score) to evaluate the reduction in dental anxiety after exposure to different anxiety‐reduction techniques. It was observed that the participants exposed to iatrosedation demonstrated a significant decrease in anxiety levels as compared with other patients. Such findings have also been reported in a study by Friedman and Wood (1998), which compared dental anxiety in patients in relation to iatrosedation and standard interviews. Furthermore, Sandhu et al. exhibited the iatrosedation technique as an effective modality in easing the anxiety of patients who could not adapt to their dentures (Sandhu et al., 2016). These findings highlight the vital role of communication between dentists and patients before dental procedures, which includes carefully explaining the entire treatment procedure, potential procedural risks, and patient preferences. It is essentially a process of re‐learning, which provides for acknowledging, exploring, interpreting, and explaining the problem to the patient and proposing possible solutions. Therefore, iatrosedation creates trust between the patient and dentist and is an indispensable tool for successful patient management. However, at times, the iatrosedation technique might not mitigate the dental anxiety of patients as it is based on treating the dental anxiety of patients based on previous traumatic experiences. Hence, combination with other anxiety‐reduction techniques can prove to be beneficial.

Music listening is known to have a relaxing effect on individuals by affecting their moods and emotions (Adiasto et al., 2022). In the present study, we evaluated the impact of music listening on alleviating dental anxiety experienced by patients. The present study concluded that music listening resulted in a decrease in the anxiety of patients when the heart rate and VRS scores of the patients were taken after the implementation of the anxiety‐reduction technique; however, the anxiety reduction was not significant. Studies have shown controversial outcomes related to the efficacy of music listening in anxiety reduction (Bradt & Teague, 2018; Moola et al., 2011). In a systematic review by Moola et al., three out of seven studies showed the anxiety‐reduction effect of music listening among patients with dental anxiety (Moola et al., 2011). The review concluded that there may be evidence of anxiety reduction in adults due to music listening; however, among children, music listening was ineffective (Moola et al., 2011). Moreover, a study by Karapicak et al. reported that music listening decreased the VRS scores of patients experiencing moderate anxiety (Karapicak et al., 2023). Such findings are consistent with the conclusions of our study. As the efficacy of music listening for anxiety reduction is debatable, the authors recommend its use in combination with relaxation and iatrosedation for effective dental‐anxiety management (Moola et al., 2011). A study by Spindler and colleagues suggested that iatrosedation may not be flexible enough for patients without traumatic backgrounds due to dental fear (Spindler et al., 2015).

In addition to music listening and iatrosedation, other techniques have been proposed to offer suitable alternatives for the anxiety reduction of dental patients. Pharmacological interventions, including the use of drugs such as midazolam and benzodiazepines, have been used for patients with moderate to severe dental anxiety (Dantas et al., 2017; Thom et al., 2000). The use of pharmacological therapy along with psychological measures such as iatrosedation can be used in geriatric patients to limit the dose of the drug required by the patient (López‐Jiménez & Giménez Prats, 2004). Moreover, behavior management, hypnosis, virtual reality, and biofeedback relaxation have also been investigated to reduce dental anxiety in patients (Padminee et al., 2022; Ramírez‐Carrasco et al., 2017). Therefore, further studies are warranted to compare the efficacy of contemporary methods with the standard iatrosedation method in managing patients with dental anxiety.

Many factors lead patients to experience different levels of distress when receiving dental treatment, with age being one of them. Patients of different ages experience different levels of dental anxiety. In this study, patients who belonged to the younger age group reported higher levels of anxiety as compared with older individuals. These findings are in line with previous studies, suggesting that younger individuals experience higher levels of dental anxiety compared with older adults (Abbasi et al., 2021; Musalam et al., 2021; Zinke et al., 2019). Since young patients are visiting the dentist for the first time, they tend to imagine every sort of painful experience that can occur during dental treatment, which can lead to greater levels of anxiety. By contrast, owing to favorable dental treatment experience in the past, older patients tend to have little or no apprehension about dentists.

Interestingly, patient's sex can affect perceived levels of dental anxiety (Lal et al., 2022). In addition, studies have shown higher levels of dental fear among females than among males (Heft et al., 2007; Lal et al., 2022). However, in the present study, there was no correlation between dental anxiety and patient's sex, in contrast to the findings of previous studies (Heft et al., 2007; Lal et al., 2022). Despite suffering from higher dental anxiety, females tend to visit the dental practice more frequently, taking better care of their oral hygiene than males (Singh et al., 2021).

In this study, iatrosedation was found to be the most effective tool in reducing the dental anxiety of patients, while music listening showed limited anxiety reduction. However, these outcomes should be interpreted in light of possible study limitations. A post hoc study power calculation showed a less than 70% power and nearly 0.4 effect size for the music‐listening group compared with the iatrosedation group (>90% power and 1.6 effect size). A larger sample size would have shown a different outcome for heart rate in the music listening group. Dental anxiety is commonly associated with children; however, the present study included only an adult population. In addition, a combination of anxiety‐reduction therapies (distraction, behavior management, drugs, and iatrosedation) has been recommended in the past for more effective dental treatment and management in anxious patients; however, individual anxiety‐reduction methods were employed in the present study. Therefore, further randomized controlled trials with improved sample sizes investigating the combination efficacy of multiple anxiety‐reduction techniques in adults and children are recommended.

## CONCLUSION

Use of the iatrosedation technique resulted in a significant reduction of dental anxiety for patients undergoing dental crown treatment; however, the use of music listening was less effective than iatrosedation. Dental anxiety is a frequently encountered problem in dental clinics that causes the avoidance of dental care. Educating patients regarding the dental care they are about to receive is vital to reducing their anxieties.

## FUNDING INFORMATION

King Saud University, Researchers Supporting Project (RSP2023R738), Riyadh, Saudi Arabia.

## CONFLICT OF INTEREST STATEMENT

The authors declare that they have no conflict of interest.

## ETHICS STATEMENT

The study was executed as per the Declaration of Helsinki. The Ethics and Review Committee of Altamash Institute of Dental Medicine, Pakistan, approved the study. All patients included were given an explanation of the study procedure, and their concerns and questions were addressed. Patients provided informed consent for inclusion in the study.

## Data Availability

The datasets used and/or analyzed during the current study are available from the corresponding author upon reasonable request.
